# Miscellany

**Published:** 1912-04

**Authors:** 


### MISCELLANY

---

### Causes of Death by Age Periods in the Registration Bureau of the U. S.

From Bulletin 109, U. S. Census, for 1910

The total number of deaths in 1910 for the 53,843,896 population was 805,412. Of these, 154,373 were infants under 1 year of age, 33,080 were 1 year old, 14,727 were 2 years old, 8,808 were 3 years old, 6,331 were 4 years old, 217,319 were under 5 years, 17,943 were 5 to 9 years old, 235,262 were under 10 years old, 31,508 were 10 to 19 years old, 62,957 were 20 to 29 years old, 68,957 were 30 to 39 years old, 72,935 were 40 to 49 years old, 81,540 were 50 to 59 years old, 96,651 were 60 to 69 years old, 96,000 were 70 to 79 years old, 51,401 were 80 to 89 years old, and 7,974 were 90 years and over.

THE IMPORTANT CAUSES OF DEATH.

Among the deaths numbering 805,412, from all causes at all ages in 1910, tuberculosis (all forms) was the most important cause, being responsible for 10.7 per cent. of the total; organic diseases of the heart followed with 9.5 per cent.; diarrhoea and enteritis, 7.8 per cent.; pneumonia (lobar and undefined 6.7 per cent.; acute nephritis, Bright's disease, 6.6 per cent.; accident (not including injuries at birth) 5.6 per cent.; cancer and other malignant tumors (all forms) 5.1 per cent.; cerebral hemorrhage, apoplexy, 4.9 per cent.; bronchopneumonia, 3.1 per cent.; premature birth, 2.5 per cent.; congenital debility, 1.9 per cent.; old age, 1.7 per cent.; typhoid fever, 1.6 per cent., bronchitis (acute and chronic 1.6 per cent.; Diphtheria and croup, 1.4 per cent.; diseases of the arteries, atherma, aneurysm, etc., 1.4 per cent.; suicide, 1.1 per cent.; and 1.0 per cent. each for influenza, diabetes, paralysis without specified cause, other diseases of the stomach (cancer excepted), the puerperal state, and malformations.

CAUSES BY AGE-PERIODS.

For infants under 1 and 1 and 2 years of age, diarrhoea and enteritis was the most important cause of death, the percentage being 29.0, 28.9, and 12.9, respectively. Diphtheria and croup caused the largest proportion of deaths of children 3 and 4 years of age, the percentage being 16.4 and 18.2 respectively. For the entire group of children under 5 years of age, the leading cause was diarrhoea and enteritis, 26.3 and for children from 5 to 9, it was diphtheria and croup 16.4. Diarrhoea and enteritis caused 24.5 per cent. of all deaths among children under 10 years of age.

TUBERCULOSIS GREATEST FROM 10 TO 50 YEARS OF AGE.

Tuberculosis caused by far the largest proportion of deaths at each 10-year age period from 10 to 50 years of age. At 10 to 19 years, it formed 24.5 per cent. of the total deaths; at 20 to 29 years, 35.0; at 30 to 39 years, 28.5; and at 40 to 49 years, 18.3. At 50 to 59, 60 to 69, 70 to 79, 80 to 89, 90 and over, the percentages were 10.2, 5.1, 2.4, 0.9 and 0.4, respectively. It formed 1.6 per cent. of all deaths among infants under 1 year of age and steadily increased to 5.3 at 1 year, 6.1 at 2 years, 6.9 at 3 years, 7.2 at 4 years, but forming only 2.8 for under 5 years, although going as high as 7.9 per cent. in the age-period from 5 to 9 years, and 3.2 for under 10 years.

HEART DISEASES RULE FROM 50 YEARS UPWARD.

Organic diseases of the heart constituted the most important cause of death at each age period between 50 and 90 years of age and also at the period 90 years and over, barring "old age."

The percentage of deaths from heart disease at the age-periods, 50 to 59, 60 to 69, 70 to 79, 80 to 89, 90 and over, was 13.4, 18.2, 19.8, 17.0, and 11.2 respectively. It caused 0.6 per cent. of all deaths among children under 1 year of age, 0.4 at 1 year, 0.7 at 2 years, 1.0 at 3 years, 1.6 at 4 years, 0.6 of all under 5 years, 4.0 of all from 5 to 9 years and 0.9 of all under 10 years of age.

BRIGHT'S DISEASE AND CANCER.

Acute Nephritis, Bright's disease, caused from 3.4 to 6.3 per cent. of the deaths at the 10 year age periods from 10 to 39 years of age, but it increased to 9.5 per cent. at 40 to 49 years, advancing again to 11.7 per cent. at 50 to 59 years, still increasing to 12.1 per cent. at 60 to 69 years, then falling to 11.1 per cent. at 70 to 79 years, decreasing again to 8.5 per cent. at 80 to 89 years, and finally becoming 5.4 per cent. at 90 years and over. It caused 0.8 per cent. of the deaths at 1 year, 1.3 at 2, 2.0 at 3, 2.2 at 4, 0.7 of all deaths of children under 5 years of age, 2.6 of all deaths at the 5 to 9 years age-period, and 0.8 per cent. of the deaths of all children under 10 years of age.

Cancer is not charged with any percentages in the age periods up to 19 years of age, but it formed 1.1 per cent. at 20 to 29 years, 3.8 per cent. at 30 to 39 years, 9.0 per cent. at 40 to 49 years, 12.1 per cent. at 50 to 59 years, 11.1 per cent. at 60 to 69 years, 7.8 per cent. at 70 to 79 years, 4.5 per cent. at 80 to 89 years, and 2.2 per cent. at 90 years and over.

TYPHOID AND APOPLEXY.

Typhoid fever caused 8.0 per cent. of the deaths at the age period 10 to 19 years but decreased to 6.0 per cent. at 20 to 29 years, to 3.2 per cent. at 30 to 39 years, to 1.8 per cent. at 40 to 49 years, to 1.1 per cent. at 50 to 59 years, there being none for the remaining age-periods. It formed 0.4 per cent. of all deaths at 1 year of age, 0.9 at 2, 1.6 at 3, 2.6 at 4, and 3.8 per cent. at the age-period 5 to 9 years of age.

Cerebral hemorrhage, or apoplexy, began its course with 0.7 per cent. of all the deaths at the 20 to 29 years age-period, then constantly increasing to 1.7 at 30 to 39 years, 4.0 at 40 to 49 years, 7.5 at 50 to 59 years, 10.7 at 60 to 69 years, 12.1 at 70 to 79 years, but falling to 10.8 at 80 to 89 years, and 7.5 at 90 years and over.

ACCIDENTS, ETC.

Accident (not including injuries at birth) claimed 0.9 per cent. of all deaths among infants under 1 year, 3.4 at 1 year, 7.3 at 2 years, 9.0 at 3 years, 10.3 at 4 years, 2.3 of all in the under 5 years age period, 12.0 of all in the 5 to 9 years, 3.1 of all deaths of children under 10 years, 13.1 at 10 to 19 years, 12.9 at 20 to 29 years, 10.5 at 30 to 39 years, 8.2 at 40 to 49 years, 5.3 at 50 to 59 years, 3.4 at 60 to 69 years, 2.9 at 70 to 79 years, 3.5 at 80 to 89 years, and 5.1 at 90 years and over.

Suicide starts with 1.1 per cent. of all deaths at the 10 to 19 years age-period, increasing to 2.8 at 20 to 29 years, then decreasing to 2.6 at 30 to 39 years, 2.3 at 40 to 49 years, 1.8 at 50 to 59 years, stopping with 1.0 per cent. of all deaths at the age period of 60 to 69 years.

Homicide begins with 1.7 per cent. of all deaths at the age period of 20 to 29 years, 1.2 at 30 to 39 years, and 0.6 at 40 to 49 years.

PNEUMONIA AND APPENDICITIS.

Pneumonia (lobar and undefined) claims 5.5 per cent. of all deaths of infants under 1 year of age, 10.1 at 1 year, 10.0 at 2 years, 8.1 at 3 years, 7.5 at 4 years, 6.6 of all deaths in the age period of under 5 years, 6.3 at 5 to 9 years, 6.6 of all under 10 years, 5.7 at 10 to 19 years, 5.6 at 20 to 29 years, 7.1 at 30 to 39 years, 7.8 at 40 to 49 years, 7.6 at 50 to 59 years, 7.1 at 60 to 69 years, 6.5 at 70 to 79 years, 5.7 at 80 to 89 years, and 5.1 at 90 years and over.

Appendicitis begins with 1.0 per cent. of all deaths of children at 4 years of age, 3.2 of all at 5 to 9 years, 4.7 at 10 to 19 years, 2.0 at 20 to 29 years, 1.4 at 30 to 39 years, and ends with 1.0 at 40 to 49 years.

---

Clean Money.—The Baltimore Clearing House is agitating for a reform in this particular. In Washington, the stranger is detected by his dirty bills. Out west, the tenderfoot betrays himself by objecting to a load of silver dollars. Bacteria are said to die upon metallic surfaces but the portability of bills renders them almost a necessity. A girl "worth her weight in gold" is only moderately well-to-do, (say $40,000) and the old tales of carrying off a fortune in gold fall flat in these days of the little dollar. We have previously suggested that aluminum, which is light and which can be both etched and perforated as a metal and printed like paper, would be an ideal base for fiat money. Cards of aluminum of the size into which a bill is usually folded, would be portable as paper money and would be amenabe to protection against raising the denomination and somewhat fireproof.

